# Hepatic encephalopathy due to extrahepatic portosystemic shunt

**DOI:** 10.1002/ccr3.2986

**Published:** 2020-06-01

**Authors:** Yosuke Sazumi, Yoshito Nishimura, Yuki Otsuka, Yuki Mizuta, Yasuhiro Nakano, Hiroyuki Sakae, Fumio Otsuka

**Affiliations:** ^1^ Department of General Medicine Dentistry and Pharmaceutical Sciences Okayama University Graduate School of Medicine Okayama Japan

**Keywords:** extrahepatic portosystemic shunt, hepatic encephalopathy, portal hypertension and altered mental status

## Abstract

Clinicians should recognize extrahepatic portosystemic shunt as a cause of refractory or intermittent hepatic encephalopathy. Treatment strategies should be individualized according to patients' anatomic and hemodynamic status.

## Graphical abstract

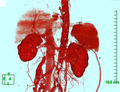

## 1 CASE

**FIGURE 1:**
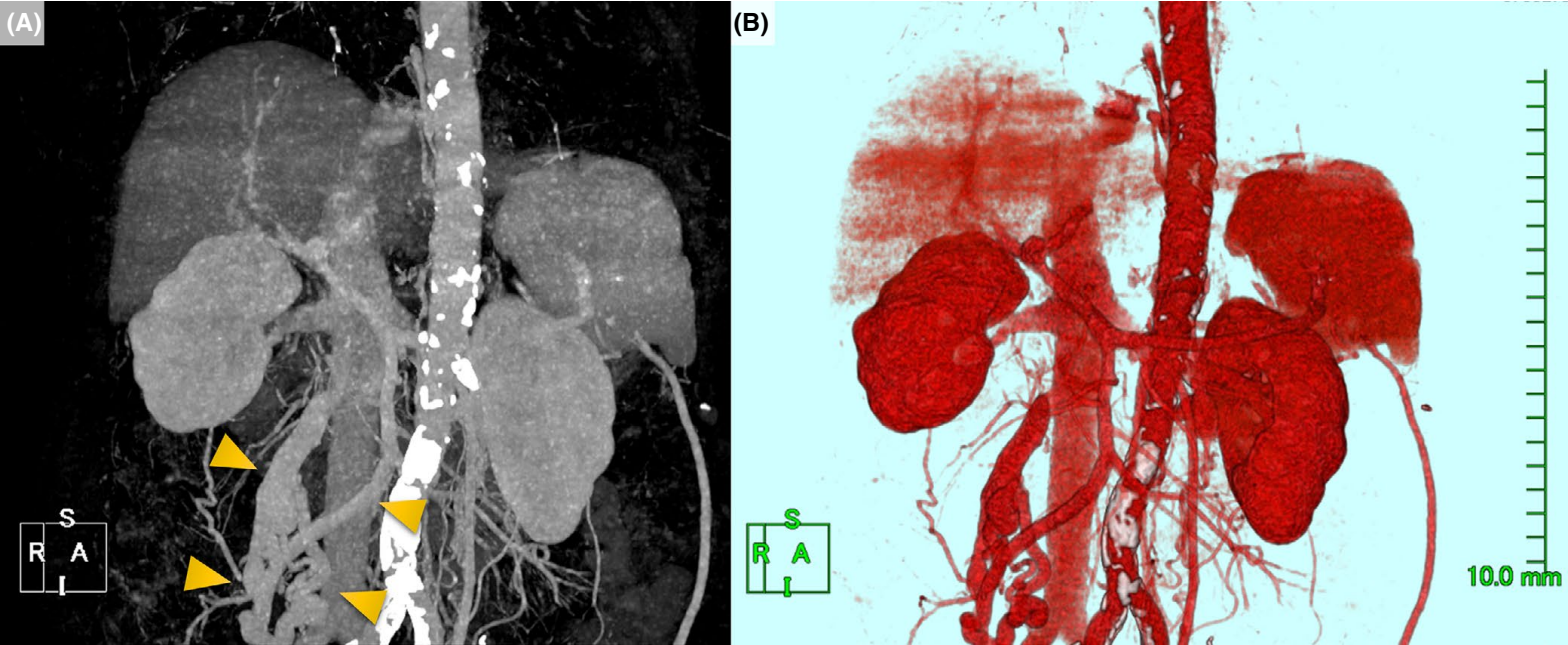
(A, B) 3D‐reconstructed computed tomography images revealing extrahepatic portosystemic shunt connecting superior mesenteric veins with inferior vena cava (arrowheads). Figure 1B depicts the vasculature only

A 70‐year‐old woman was admitted to our hospital with a 2‐week history of intermittent altered mental status. She had known cirrhosis due to alcoholic hepatitis, but she had not had any similar symptoms in the past. Physical examination revealed asterixis, but no hepatomegaly and caput medusae were noted. Laboratory findings were significant for hyperammonemia without elevated liver transaminase levels. Electroencephalogram revealed triphasic waves suggestive of hepatic encephalopathy. Abdominal computed tomography (CT) revealed extrahepatic portosystemic shunt (EPSS) characterized by direct communication of the dilated superior mesenteric veins (SMV) with the inferior vena cava (IVC) Figure 1A and B. The shunt was considered to be secondary to portal hypertension and large,therefore, combination therapy with lactulose and rifaximin was initiated, which improved her mental status. She has been followed up on outpatient basis by a gastroenterologist with the combination therapy.

Extrahepatic portosystemic shunt is a vascular anomaly where venous blood from the intestines directly returns to the systemic circulation bypassing the liver. While EPSS occurs due to a congenital anomaly, it may be idiopathic or the consequence of portal hypertension. According to a retrospective study, the incidence of SMV‐IVC shunt was rare, accounting for only 2% of all EPSS cases.^1^ Although shunt occlusion should be considered in these cases, patients with large shunts, as of this case, have a high risk of drawbacks including esophagogastric varices and intractable ascites due to increased portal vein pressure.^2^ Thus, therapeutic strategies should be individualized according to patients' anatomic and hemodynamic status. Clinicians should recognize EPSS as an unusual cause of intermittent hepatic encephalopathy.

## CONFLICT OF INTEREST

The authors declare no conflicts of interest.

## AUTHORS' CONTRIBUTION

YN and YS: wrote the first draft and managed all the submission process. YO, YM, YN, HS, and FO: contributed to the clinical management of the patients and revised the manuscript.
